# Effects of systemic administration of HESA-A on the expression of cyclin D1 and EGFR and E-cadherin in the induced tongue dysplasia in rats

**DOI:** 10.15171/joddd.2017.036

**Published:** 2017-12-13

**Authors:** Shirin Fattahi, Sepideh Vosough Hosseini, Amir Ala Aghbali, Masoumeh Mehdipour, Sanaz Helli, Hossein Damghani

**Affiliations:** ^1^Department of Oral and Maxillofacial Pathology, Faculty of Dentistry, Tabriz University of Medical Sciences, Tabriz, Iran; ^2^Department of Oral Medicine, Faculty of Dentistry, Shahid Beheshti University of Medical Sciences, Tehran, Iran

**Keywords:** Biomarkers, cadherin, carcinoma, EGFR protein, squamous cell carcinoma, rat

## Abstract

***Background.*** HESA-A has herbal and marine bases, containing minerals and rare elements such as Zr, Cr, Ga, Mn, Mg, Ca, Sr, Cu, Ti, etc. Its mechanism of action includes antioxidant, antiinflammatory and adjustment of the immune system. The aim of this study was to evaluate the effects of HESA-A systemic drug on expression of cyclin D1, EGFR and E-cadherin in induced tongue dysplasia in rats.

***Methods.*** In this experimental study, the effects of the systemic drug HESA-A on the expression of cyclin D1, EGFR, and E-cadherin molecular markers were examined in induced tongue dysplasia in rats.

***Results.*** The incidence rate of cyclin D1 in groups receiving HESA-A was lower than the group that did not receive the drug (77.78% in the 0‒5% range versus 77.78% in the 5‒50% range). In the case of expression of E-cadherin in group D, which did not receive HESA-A, a decrease was observed in the expression of this cell adhesion marker as compared to the other two groups. The incidence of E-cadherin was dependent on HESA-A dose, while with 500 mg/kg it was higher than other groups (>75% in 55.55% versus >75% in 11.11%). Concerning the incidence of EGFR in all the three groups most cases were grade 0.

***Conclusion.*** The results of the present research indicated that considering changes in the expression of cyclin D1 and E-cadherin markers in groups treated with HESA-A, HESA-A® has preventive effects on development of cancer in dysplastic lesions through regulation of expression of these molecules.

## Introduction

Oral cancer includes a variety of malignant neoplasms in the oral cavity and it is commonly found on the tongue, oropharynx and floor of the mouth. Squamous cell carcinoma (SCC) comprises 90% of malignant tumors of the oral cavity. It is derived from the oral squamous epithelial cells and refers to the abnormal and uncontrolled growth of squamous epithelial cells which are also affected by changes in tissue and cell differentiation such as keratinization and changes in tonofilament bundles and desmosomes related to cellular connections.^1^

The incidence of SCC is a multifactorial process occurring due to the accumulation of genetic and environmental factors. Risk factors for oral cancers include excessive smoking and alcohol consumption, lack of fruits and vegetables in the diet, chewing tobacco, genetics, some viruses, a family history of oral cancer or other types of cancer and pre-malignant lesions.^2^

The causative factors of SCC are multiple processes occurring due to the cumulative effects of environmental and genetic factors over the years, resulting in mutations and oncogenic changes‏.

Recently, many studies have focused on the overexpression of cyclin D1 proto-oncogene (cell cycle regulatory protein) and EGFR (growth factor of epithelial cells) in cancers and the literature in this field is quite abundant.^3^

In some studies, it has even been suggested that overexpression of both these markers leads to worse clinical outcomes (such as higher stage of cancer and lower prognosis).^4^ Recently FDA has reported EGFR inhibitors among cancer treatments.^5^

E-cadherin is a cell adhesion molecule expressed in most normal epithelial tissues. This molecule is correlated with the formation of the glands and epithelial differentiation and polarity. Selective loss of E-cadherin would lead to a lack of differentiation and invasion in human carcinomas.^6,7^

Natural ingredients contain antioxidants which can fight cancer cells. Antioxidants protect the body against damage, especially against damage caused by free radicals, and prevent the growth of cancer cells and tumors. The biological nature of traditional medicine results in greater consistency with the body and elimination of side effects. Today, traditional medicine, especially herbal medicine, is widely used to prevent and treat diseases.^8^

Regarding the climatic conditions, Iran enjoys a vast and unique biodiversity which is mostly based on the use of herbal medicines and natural resources. One of these drugs produced in our country is HESA-A, which has a herbal and marine origin and contains minerals and rare elements that act on the basis of antioxidant, antiinflammatory and immunomodulatory mechanisms with the induction of apoptosis.^9^

HESA-A bears selective effects, i.e. it has no effect on normal cells and is categorized as a drug with No-Observed-Adverse-Effect Level (NOAEL). The drug has been approved by the Ministry of Health and is manufactured by one of the pharmaceutical companies as coated tablets.^10^

Previous studies which were carried out on HESA-A were mostly associated with gastrointestinal system, breast and bone cancers. ^11-13^

Except for the aforementioned research study (Mehdipour et al), no study has been conducted on tongue cancer. Previous in vitro or in vivo studies have had many shortcomings and such shortcomings are more prominent with in vitro studies. They included more difficult control of confounding factors in the study, errors related to study inclusion and exclusion criteria, ethical considerations, unexpected adverse drug reactions during human studies and much higher costs‏.^12^

Despite the effect of HESA-A on cancerous markers of tongue cancer or dysplasia, no study has been conducted in this field; therefore, this in vitro study was undertaken to investigate its effects for the first time, using the pre-prepared paraffin blocks.^13^

According to the results of an in vitro study carried out by Mehdipour et al in Tabriz Faculty of Dentistry, HESA-A has a positive role in preventing cancer in rat tongue. The result was obtained based on histopathological examinations on the autopsy of animals' tongue stained with H&E and dysplastic changes of samples‏. Furthermore, the group receiving a higher dose of HESA-A (group B) showed a lower degree of dysplasia compared with those receiving a lower dose of HESA-A (group C). This reflects the fact that the anticancer effects of HESA-A are dose-dependent and its effects increase as the dose increases.^14^

As mentioned in the previous research, over-expression of EGFR and cyclin D1 molecular markers is associated with cancer. This means that their overexpression in the premalignant lesions of the oral cavity increases the risk of cancer.^15^

In the study carried out by Mehdipour et al, investigations were solely carried out on the extent of dysplasia with regard to histopathological changes obtained from H&E staining, and the molecular markers involved in cancer were ignored. Their study was conducted for the first time with a focus on tongue cancer and HESA-A among the research community.^14^

Since no study has been conducted on the effects of the anti-cancer drug HESA-A on the expression of molecular markers involved in tongue cancer and since these molecular markers are of paramount importance in the early diagnosis of tongue cancer and pre-malignant lesions, we investigated the effect of HESA-A on reducing the expression of the above-mentioned molecular markers.

## Methods

In this experimental study, the effects of the systemic drug HESA-A on the expression of cyclin D1, EGFR and E-cadherin molecular markers in rats' induced tongue dysplasia were examined‏.

The study population consisted of paraffin blocks obtained from the previous study‏. In the previous study, 48 microscopic plates were prepared from the dead rats and used to examine dysplasia in H&E stained sections. In this study, the incidence of SCC was positive in 83% of cases in the group receiving 4NQO carcinogenic drug.

In previous study, four groups were considered as follows‏:

Group A: 12 rats as a control group treated with normal saline solution three times a week
Group B: Adding 4-nitroquinoline oxide at a concentration of 30 ppm to animals' water for 12 weeks along with simultaneous treatment with 500 mg/kg of HESA-A three times a week
Group C: Adding 4-nitroquinoline oxide at a concentration of 30 ppm to animals' water for 12 weeks along with simultaneous treatment with 250 mg/kg of HESA-A three times a week
Group D: Adding 4-nitroquinoline oxide at a concentration of 30 ppm to animals' water for 12 weeks without adding drugs, treated with normal saline solution three times a week

In this study the control group was removed and 28 paraffin blocks were used in the project.

In this study, the incidence of the cancer-involved markers was compared between the groups receiving carcinogens simultaneously with HESA-A and those who only received carcinogen (Group D).

As it was mentioned before, the study inclusion criteria involved all the paraffin blocks of groups B, C and D, which had sufficient tissues to be cut. It should be noted that all the exclusion criteria as well as all interfering cases had been omitted or taken under control in the previous study‏.

The blocks of different groups were separately cut and underwent immunohistochemistry staining‏.

In order to have immunohistochemical examination, 3 cuts of paraffin blocks measuring 3 µm in length were prepared and underwent immunohistochemistry staining based on the DAKO Company Protocol‏.

The total number of cuts was 84. According to previous studies, the following scores were given: Four scores for EGFR^16^ and E-Cadherin^17^ staining and 3 scores for Cyclin D1 staining.^18^ Data were subjected to statistical analyses.

## Results

In this study, the effects of the systemic drug HESA-A were examined on the expression of cyclin D1, EGFR and E-cadherin molecular markers in rats' induced tongue dysplasia in three groups and the following results were obtained.

### Cyclin D1 (Figure 1)

Group B: 7 cases were in the range of 0‒5% and 2 cases were in the range of 5‒50%‏.

Group C: 6 cases were in the range of 0‒5% and 4 cases were in the range of 5‒50%‏.

Group D: 2 cases were in the range of 0‒5% and 7 cases were in the range of 5‒50%‏.

**Figure 1:**
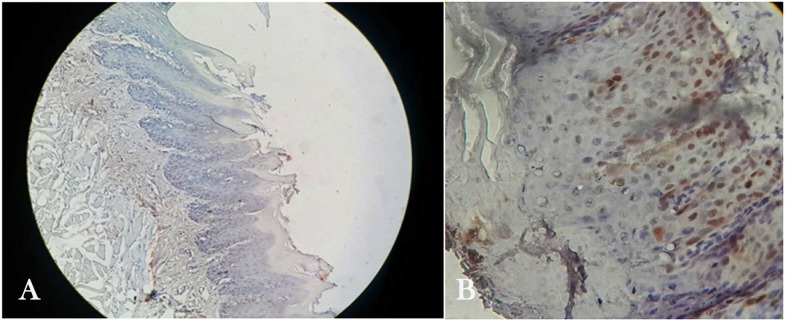
Cyclin D1 immunohistochemical staining in tongue dysplasia (A and B) (A: grade 0: non-staining of cells. B: grade 1: staining 5‒50% of cells) (×400).

### E-cadherin

Group B: There were 2 cases in the range of 1‒50%, 2 cases in the range of 51‒75% and 5 cases in the range of >75%.

Group C: There were 2 cases in the range of 1‒50%, 5 cases in the range of 51‒75% and 3 cases in the range of >75%.

Group D: There were 7 cases in the range of 1‒50%, 1 case in the range of 51‒75% and 1 case in the range of >75%.

### EGFR

Group B: There were 9 cases below the range of 10% (grade 0)‏.

Group C: There were 9 cases below the range of 10% (grade 0) and 1 case in the range of 10‒25% (grade 1^+^)‏.

Group D: There were 9 cases below the range of 10% (grade 0) and 1 case in the range of 10‒25% (grade 1^+^)‏.

## Discussion

Currently, there have appeared new tendencies toward improving the life quality of cancer patients, using traditional medicine and medical supplements so that the potential benefits of the complementary and alternative therapies are employed in cancer patients with a high probability of success. However, using the benefits of traditional and complementary treatment methods does not indicate their replacement with standard treatments. Instead, the traditional treatment methods and herbal drugs play the role of adjunctive treatment in this case. In other words, the goal of using traditional medicine and complementary modality is to support standard treatments and to provide the patients with the potential benefits in the treatment of invasive and destructive cancers but not to replace conventional treatments. In this context, the results of some studies and clinical trials have demonstrated the efficacy of traditional and complementary medicine in the treatment of cancer and improving the life quality of cancer patients.

HESA-A is a natural product of marine and herbal origin produced in Iran. Its antineoplastic properties have been confirmed under clinical and laboratory conditions.^11^

It seems that its use has some selective effects on tumor cells. In other words, this drug targets tumor cells with no damage to normal cells. Moreover, the cytotoxicity of HESA-A has also been examined and it has been determined that the drug has no toxic effects on normal cells.^19^

HESA-A contains rare elements and factors such as selenium, strontium, chromium, zinc and molybdenum. Low cytotoxicity of HESA-A may be due to its rare elements and components with low toxicity and even the antioxidant properties of some components such as selenium.

In several studies using herbal-marine HESA-A in the prevention of cancers in laboratory and clinical conditions, some promising results have been recorded.^10^

Sadeghi Aliabadi and Ahmadi (2003) reported that HESA-A in therapeutic doses and in a dose-dependent manner was specifically more effective in preventing the growth of cancer cells compared with normal cells.^20^

Moreover, Sadeghi Aliabadi et al examined the selective toxic effects of HESA-A biological composition on cancer and normal cells and claimed that this drug can selectively prevent the growth of cancer cells. This property of the drug is dependent upon its concentration.^21^

Ahmadi et al (2005) reported the effects of retreatment with HESA-A for the liver damage induced by thiacetamide in rabbits. Pretreatment with HESA-A has yielded protective effects against liver damage.^19^

Roudkenar et al (2012) also showed that in the presence of HESA-A toxicity significantly reduced and based on the results of antioxidants analysis, HESA-A could eliminate free radicals.^22^

**Figure 2:**
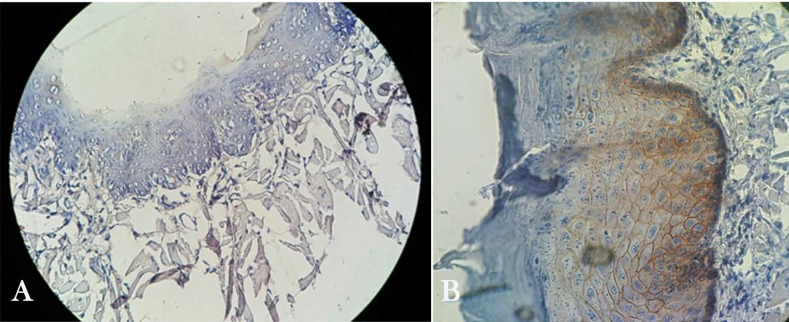
E-Cadherin immunohistochemical staining in tongue dysplasia (A and B) (A: grade 1: staining 1‒50% of cells. B: grade 3: staining more than 75% of cells) (×400).

**Figure 3:**
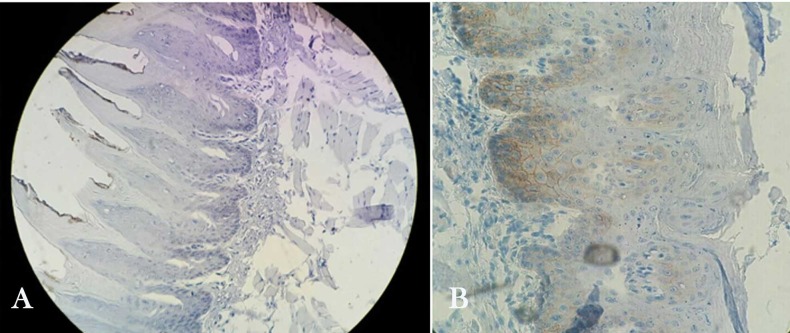
EGFR immunohistochemical staining in tongue dysplasia (A and B) (A: grade 0: staining less than 10% of cells. B: grade 1: staining 25‒10% of cells) (×400).

**Chart 1‏:**
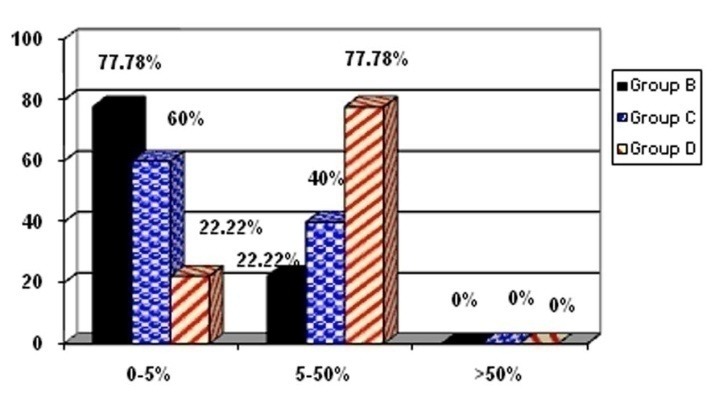
Results of Cyclin D1 between the three groups (%).

**Chart 2.**
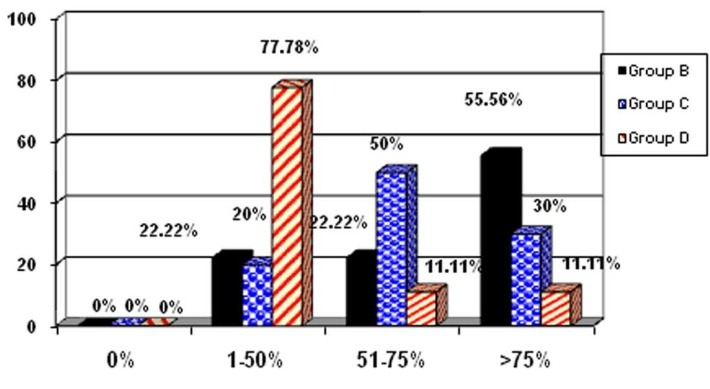
Results of E-Cadherin between the three groups (%).

**Chart 3:**
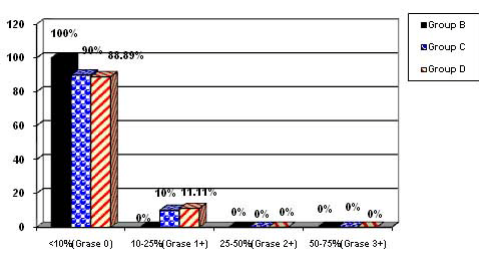
Results of EGFR between the three groups.

Ahmadi et al (2009) examined the results of treatment with HESA-A (at a dose of 50 mg/kg/d divided into two to three doses) in patients with metastatic colon cancer for six months and confirmed its benefits.^12^

In the same manner, Ahmadi et al (2010) treated thirty patients with liver metastases and final-stage cancer using the HESA-A for three months (with 50 mg/kg/d dose divided into two to three doses) and reported that HESA-A is a safe and effective anti-cancer composition and can increase the survival rate of cancer patients in final stages.^23^

In another study conducted by Jahanban et al (2015), in the Department of Biotechnology of Tabriz University of Medical Sciences, HESA-A was found to have anti-cancer and anti- cytotoxic properties.^24^

In the study by Mahdipour et al, administration of HESA-A resulted in a decrease in the severity of dysplastic lesions and more reduction in the intensity of dysplastic lesions was recorded by increasing the prescribed dose of HESA-A in animal samples. On the other hand, differences between these groups were statistically significant, and it seemed that HESA-A herbal-marine compound had preventive effects on the incidence of dysplasia and its effects were dose-dependent. Therefore, its preventive effects increased with an increase in the prescribed dose.^14^

In view of the positive contribution of HESA-A to the decrease in tongue dysplasia in Mehdipour’s study, and considering the fact that in that study the intensity of dysplasia was measured through hematoxylin staining, in this study the more specific immunohistochemistry method was used for more precise examination of the effects of this drug. In this study, expression of E-cadherin, EGFR and cyclin D1 markers, whose changes in oral dysplasia have been confirmed, was investigated.

Over-expression of cyclin D1 and EGFR proto-oncogenes has been reported in different types of cancer,^3^ and in more recent studies EGFR inhibitors have been used for cancer treatment.^5^

E-cadherin is a cell adhesion molecule, whose deficiency leads to a lack of distinction and invasion in different carcinomas.^6,7^ Moreover, over-expression of cyclin D1 and EGFR molecular markers is related to cancer and intensification of dysplasia, while over-expression of these markers increases the risk of cancer in premalignant lesions of the oral cavity.^15^

In a study conducted by Abbasi et al in the Medical Research Center of Tabriz University of Medical Sciences in 2009, the effect of HESA-A on the prognosis of oral cancers was examined and it was reported that the use of oral HESA-A reduces the expression of P53 in patients as the rates of P53 were 53.4% and 13.6% in patients receiving doses of 250 and 500 mg/kg, respectively.^25^

In a study by Abbasi et al in the Medical Research Center of Tabriz University of Medical Sciences in 2015 on the effects of HESA-A on oral cancer and its prognosis, it was shown that the *erb/b2* level in patients after using HESA-A decreased from approximately 30% to 24.1% (250 mg/kg) and 3.4% (500 mg/kg).^26^

In the present study, the incidence rate of cyclin D1 in groups receiving HESA-A was lower than the group that did not receive the drug (77.78% in the 0‒5% range versus 77.78% in the 5‒50% range), reflecting the effect of HESA-A on decreasing cyclin D1 oncogene.

Expression of E-cadherin in group D, which did not receive HESA-A, decreased as compared to the other two groups. The incidence of E-cadherin was dependent on HESA-A dose, while with 500 mg/kg it was higher than other groups (>75% in 55.55% versus >75% in 11.11%).

Concerning the incidence of EGFR in all the three groups, most cases were grade 0. It was 100% in the <10% range (grade 0) in the experimental groups versus one case in the 10‒25% range (grade 1^+^) in the control group.

Despite these reports, to draw a general conclusion, it is necessary to design controlled and prospective clinical trials using large samples with long-term follow-up periods to accurately determine the mechanisms of the HESA-A and to evaluate its long-term effects on the survival and life quality of cancer patients as well as their possible side effects. Furthermore, numerous animal studies are required in order to determine the optimal dose of this drug for prevention of oral cancers in animals and to use it in the probable human research studies.

On the other hand, according to a report on the possibility of fetal toxicity at 400 mg/kg doses of HESA-A in pregnant mice in a dose-dependent manner, caution should be exercised in the prescription of this drug.^27^

The effect of HESA-A on the expression of other genes involved in oral cancer carcinogenesis should be assessed. The effects of prescription of HESA-A on human samples should be evaluated as a complementary treatment along with standard treatments.

## Conclusion

The results of the present research indicated that considering changes in the expression of cyclin D1 and E-cadherin markers in groups treated with HESA-A, HESA-A has preventive effects on the development of cancer in dysplastic lesions through regulation of expression of these molecules.

## Acknowledgments

None.

## Authors’ contributions

The study was designed by SVH and MM. The literature review was performed by AAA and SF. The immunohistochemical evaluation was carried out by SVH, SF and HD. SH and HD drafted the manuscript. All the authors contributed to the revision and final approval of the manuscript.

## Funding

This study was supported and funded by Tabriz University of Medical Sciences.

## Competing interests

The authors declare no competing interests with regards to the authorship and/or publication of this article.

## Ethics approval

The study protocol was approved by the Ethics Committee of Tabriz University of Medical Sciences.
